# Joint association between tobacco smoke exposure and periodontitis and glycemic status

**DOI:** 10.3389/fendo.2025.1539955

**Published:** 2025-04-07

**Authors:** Xiaoguo Hua, Jiong Li, Rui Hu, Xiujun Zhang

**Affiliations:** ^1^ Office of Medical Insurance Management, The Second Affiliated Hospital, Anhui Medical University, Hefei, China; ^2^ Department of Epidemiology and Biostatistics, School of Public Health, Anhui Medical University, Hefei, China; ^3^ College and Hospital of Stomatology, Anhui Medical University, Key Laboratory of Oral Diseases Research of Anhui Province, Hefei, China; ^4^ Department of Clinical Teaching Management, The First Affiliated Hospital, Anhui University of Traditional Chinese Medicine, Hefei, China

**Keywords:** joint association, tobacco smoke exposure, periodontitis, glycemic status, management

## Abstract

**Objective:**

Both tobacco smoke exposure (TSE) and periodontitis were independently associated with glycemic status. However, studies focusing on the co-exposure and relative contributions of TSE and periodontitis to glycemic status are rare. This study intended to examine the joint and mutual associations between TSE and periodontitis and glycemic status among American adults.

**Methods:**

Data were extracted from the National Health and Nutrition Examination Survey (NHANES, 2009-2014). Weighted logistic regression models were used to calculate their odds ratio (OR) and corresponding confidence interval (95% CI) with adjustments for confounding factors in the multivariate analysis that assessed the joint and mutual association between TSE and periodontitis and prediabetes and diabetes mellitus (DM).

**Results:**

TSE and periodontitis were shown to be independently associated with prediabetes, and a positive association between periodontitis and DM was observed. Significant associations between both DM and prediabetes and the joint effect of periodontitis and TSE were detected. Moreover, a positive association between periodontitis and the risk of prediabetes and DM was observed in both active and passive smokers. Among the participants with TSE, a significantly higher risk of prediabetes or DM was found in those with moderate or severe periodontitis.

**Conclusions:**

TSE and periodontitis synergistically increased the risk of incident DM or prediabetes, and the deleterious effect of periodontitis on glycemic control could be reduced by smoking abstinence. The findings highlight the importance of avoiding constant exposure to tobacco smoke or quitting smoking for the management of the glycemic status of patients with moderate or severe periodontitis.

## 1 Introduction

Globally, diabetes mellitus (DM) continues to represent a significant public health challenge, affecting more than 285 million people and imposing considerable economic burdens (1). Effective management of diabetes requires meticulous control of blood glucose levels, a task complicated by numerous factors including lifestyle choices, environmental exposures, and comorbidities (2). Among these factors, tobacco smoke exposure (TSE) (3) and periodontitis (4) emerge as modifiable risk factors that have been implicated in the modulation of glycemic status.

According to the latest estimates from the Global Burden of Disease Study 2019, the number of prevalent cases of severe periodontitis reached over 1.1 billion globally (5). There is a bidirectional relationship between periodontitis and diabetes. It has been shown that periodontitis is associated with glycemic control indicators, such as HbA1c, in prediabetic and diabetic patients (6). Previous studies have suggested that the risk of developing periodontitis is increased 2–3 times in individuals with diabetes compared to individuals without diabetes (7). A systematic review and meta-analysis also showed that periodontitis was associated with higher HbA1c levels in people who did not have diabetes, while the evidence to support these findings was weak in people with type 2 diabetes mellitus (T2DM) (8).

TSE, a complex mixture of thousands of chemicals released by the burning end of a cigarette or exhaled by smokers, has been linked to the risk of developing periodontitis and elevated blood glucose. A meta-analysis (9) of 88 prospective cohort studies confirmed a significant association between smoking and the risks of T2DM, with a relative risk of 1.37 in smokers and 1.14 in former smokers compared to never-smokers. Smoking limits the delivery of nutrients and oxygen to periodontal tissues, suppresses immune responses, and alters the oral microbiota (10), which contributes to the progression of periodontitis. Despite quitting smoking showing clear benefits in terms of glycemic control, the prevalence of smoking in people with DM seems to be comparable to that of the general population (11). Cotinine, the primary metabolite of nicotine, serves as a biomarker for TSE in active and passive smokers. The level of cotinine in the bloodstream can be used to determine the extent of TSE. Based on the fact that TSE increases the risk of periodontitis and that the combined harmful effects of them can exacerbate impaired insulin signaling and insulin resistance, this study evaluated the effect of the interplay of TSE and periodontitis on glycemic control.

## 2 Methods

### 2.1 Study design and participants

**Figure 1:**
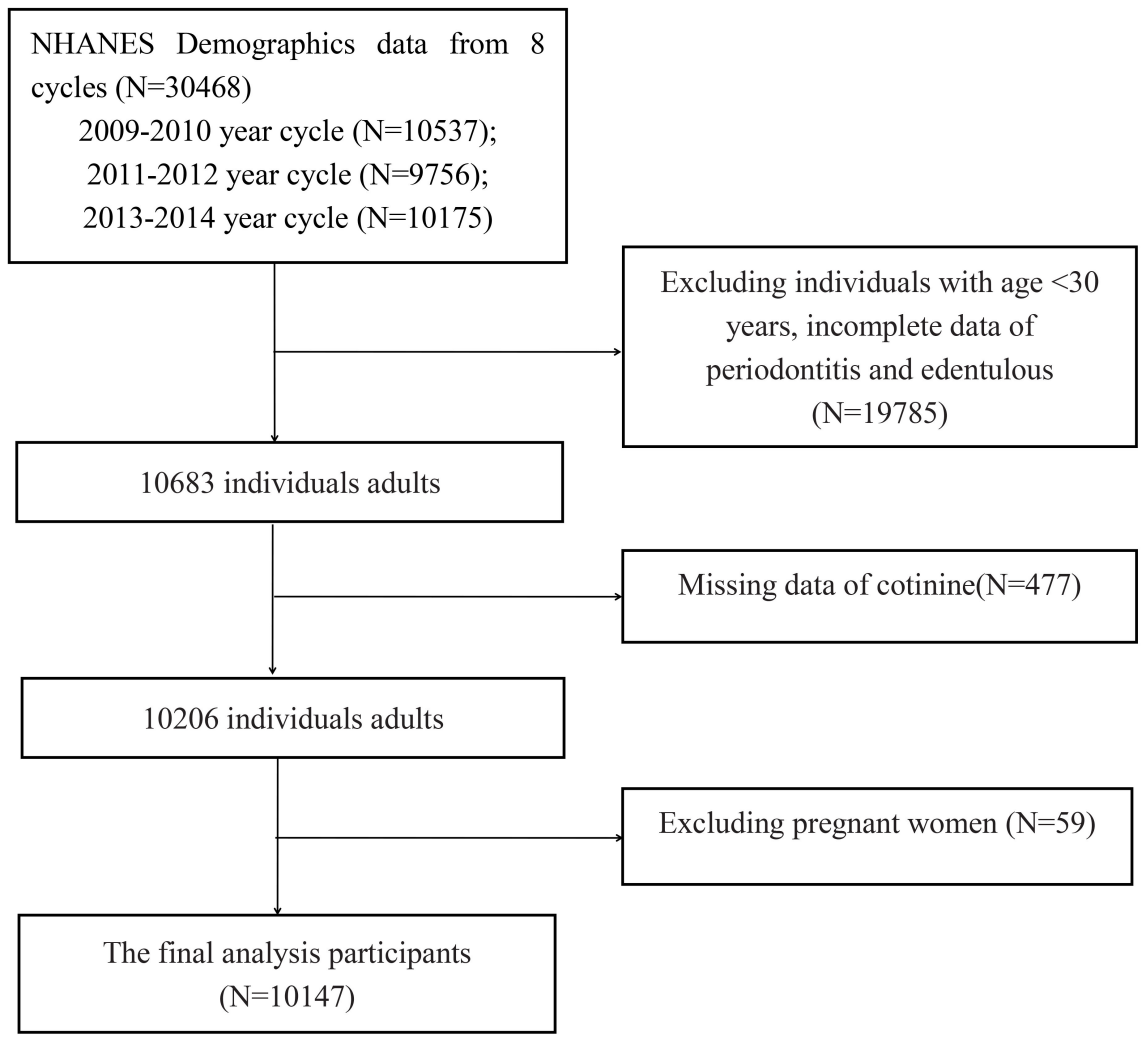
Flowchart presenting the process of study selection in detail.

This study used data from the National Health and Nutrition Examination Survey (NHANES) (https://www.cdc.gov/nchs/nhanes/index.htm). NHANES used a stratified multistage sampling design that collected multiple biological samples from adults and children in the United States. The investigation encompasses interviews, physical examinations, and laboratory tests. Additional information regarding the entire NHANES procedure and documentation is available on the official website. Study procedures were approved by the National Center for Health Statistics Research Ethics Review Board, and informed consent from all participants was provided prior to the survey. Data of participants who had periodontitis and were edentulous from three cycles (2009-2010, 2011-2012, and 2013-2014) from the NHANES database were used in our study. We extracted demographic, examination, laboratory, and questionnaire data from the NHANES for analysis in this study. Initially, there were 30,468 participants from the abovementioned NHANES cycles. The following individuals were excluded: (1) age younger than 30 years; (2) pregnant women; (3) incomplete cotinine, periodontitis, and edentulous data. A total of 10,147 adults were included for the final analysis (**Figure 1**).

### 2.2 Exposure and outcome variables

Participants meeting the Centers for Disease Control and Prevention and American Academy of Periodontics definitions were considered as having periodontitis (12, 13). Following a series of assessments according to a previous study (14), the periodontitis group was categorized into mild, moderate, or severe periodontitis, and those without periodontitis were assigned to the no periodontitis group. Serum cotinine assessments have been meticulously illustrated in our previous study (15), and a concentration less than 0.05 ng/mL was considered as TSE. The main outcomes were the risk of prediabetes or DM. DM was defined as fasting plasma glucose (FBG) ≥126 mg/dL, or HbA1c ≥6.5%, or 2 h post-load plasma glucose (PG) ≥200 mg/dL, or currently using antidiabetic medications. Prediabetes was defined as FPG 100–125 mg/dL, or HbA1c 5.7%–6.4%, or 2 h post-load PG of 140–199 mg/dL (16).

### 2.3 Covariates

After the relevant articles were retrieved and deeply evaluated, we selected the following confounding factors as covariates: age, sex (male or female), education (<high school, high school, >high school), race (Mexican American, non-Hispanic white, non-Hispanic Black, other Hispanic, or other), family poverty-income ratio (PIR; <1 or ≥1), alcohol consumption, body mass index (BMI), total cholesterol (TC), triglyceride, hypertension, dentition status, dental floss, and medication use. Alcohol consumption was divided into never (<12 drinks lifetime), mild drinker (<1 drink per week last 12 months), moderate drinker (<7 drinks per week last 12 months), and heavy drinker (≥7 drinks per week last 12 months). Hypertension was defined in patients with systolic blood pressure (SBP) ≥140 mm Hg and/or diastolic blood pressure (DBP) ≥90 mm Hg, or currently using antihypertensive medications. Dental floss was assessed by the inquiry “How many days do you use dental floss/device?”. Participants who did not use it on any day during a week were marked as “No”. The determination of whether an individual’s dentition was functional hinged on the presence of functional teeth. Specifically, possessing 20 or more natural permanent teeth was deemed to represent functional dentition, while having 19 or fewer signified non-functional dentition.

### 2.4 Statistical analyses

“WTSAF2YR” is the sample weighting code for the NHANES sample in every cycle, which was taken into account when calculating all estimates in this study. Participants were divided into four groups based on co-exposure to TSE and periodontitis. The following four groups were generated: no TSE with no periodontitis (reference group), no TSE with periodontitis, TSE with no periodontitis, and TSE with periodontitis. The Chi^2^-test and analysis of variance (ANOVA) test were used to test for differences between the four groups. The characteristics of the subjects were expressed as means and standard deviations (SDs) for continuous variables or unweighted frequencies (%) for categorical variables.

Weighted logistic regression models were used to calculate their odds ratio (OR) and corresponding confidence interval (95% CI) with adjustments for confounding factors in the multivariate analysis that assessed the joint association between TSE and periodontitis and prediabetes and DM. Outcomes were presented as Model 1 (unadjusted), Model 2 (adjusted for age and sex), Model 3 (adjusted for age, sex, race, education, BMI, alcohol intake, family PIR, TC, triglyceride, hypertension, dentition status, and dental floss). In addition, we evaluated the effect of periodontitis on the risk of prediabetes and DM stratified by TSE, and vice versa.

Subgroup analyses were conducted and stratified by age (age < 60 and age ≥ 60), sex (male or female), BMI (BMI< 25 and BMI≥ 25), and hypertension (no and yes). Sensitivity analyses were performed to assess the robustness of our primary results. First, medication use was concurrently added into the weighted logistic regression models as a covariate. Second, we performed the analysis to evaluate the associations using the complete dataset without multiple imputations and the dataset with multiple imputed analyses. Third, based on serum cotinine concentrations more than 10 ng/mL, the cut-off distinguishing smokers from non-smokers based on the NHANES evaluation, the analysis was performed by splitting TSE into three levels: No TSE (≤0.5 ng/mL serum cotinine), passive smoking (>0.5 ng/mL and ≤10 ng/mL serum cotinine), and active smoking (>10 ng/mL serum cotinine).

All analyses were performed using R software version 4.4.3 (http://www.R-project.org). A two-tailed *P*-value of < 0.05 was considered statistically significant. The visualization of the results was created using the ‘ggplot2’ package. The effect of the interplay of TSE and periodontitis was calculated using the ‘effects’ package.

## 3 Results

### 3.1 Characteristics of all participants

A total of 10,147 participants in NHANES from 2009 to 2014 were enrolled in this study, with a mean (SD) age of 50.91 (13.41) years. There were 5,042 (49.35%) men and 5,105 (50.65%) women. Non-Hispanic white participants accounted for 69.19%. **Table 1** shows the comparison of characteristics among four groups of participants cross-stratified by TSE and periodontitis. In total, 1,433 (14.12%) participants were exposed to TSE solely, 3,059 (30.15%) participants had periodontitis without TSE, and 2,843 (28.02%) participants had both periodontitis and TSE. Compared to participants with no TSE and no periodontitis, those with TSE and periodontitis were more likely to be older, male, have a low level of education, and have prediabetes and DM. Additionally, the levels of SBP, DBP, HbA1c, triglycerides, TC, and FBG were higher, and the proportion of family PIR≥1, BMI≥25 kg/m^2^, moderate or above drinking, hypertension, use of antihypertensive agents, and dental non-function was higher.

**Table 1:** Characteristics of 10,147 participants according to TSE and periodontitis.

Characteristics	Overall	No TSE and no periodontitis	Periodontitis and no TSE	TSE and no periodontitis	TSE and periodontitis	*P*
	10,147	2,812	3,059	1,433	2,843	
Age (years)	50.91 ± 13.41	49.04 ± 12.82	57.55 ± 13.66	43.93 ± 11.11	50.76 ± 12.09	< 0.001
Systolic blood pressure (mmHg)	122.59 ± 16.93	119.65 ± 15.13	126.60 ± 17.99	119.61 ± 14.59	124.41 ± 18.40	< 0.001
Diastolic blood pressure (mmHg)	72.07 ± 11.73	71.87 ± 10.83	71.22 ± 12.17	72.95 ± 11.36	72.73 ± 12.65	< 0.001
Glycated hemoglobin (HbA1c, %)	5.69 ± 0.94	5.54 ± 0.75	5.83 ± 1.02	5.53 ± 0.77	5.85 ± 1.13	< 0.001
Triglyceride (mg/dL)	159.44 ± 139.64	147.58 ± 110.18	166.71 ± 174.60	159.26 ± 123.82	169.13 ± 143.52	< 0.001
Total cholesterol (mg/dL)	197.54 ± 40.96	197.04 ± 39.30	198.01 ± 41.41	198.42 ± 40.97	197.20 ± 42.83	0.802
Fasting plasma glucose (mg/dL)	101.32 ± 35.50	97.31 ± 29.52	105.82 ± 40.77	97.25 ± 29.39	104.92 ± 39.73	< 0.001
Sex, n (%)						< 0.001
Male	5042 (49.35)	1,009 (39.05)	1,612 (52.58)	642 (46.83)	1,779 (62.67)	
Female	5105 (50.65)	1,803 (60.95)	1,447 (47.42)	791 (53.17)	1,064 (37.33)	
Race, n (%)						< 0.001
Mexican American	1,469 (8.11)	322 (5.97)	647 (12.28)	125 (5.20)	375 (8.49)	
Other Hispanic	1,007 (5.31)	295 (4.94)	397 (7.10)	113 (4.59)	202 (4.32)	
Non-Hispanic white	4,421 (69.19)	1,445 (77.07)	1,138 (64.06)	725 (71.69)	1,113 (61.59)	
Non-Hispanic Black	2,034 (10.22)	359 (5.78)	492 (8.26)	321 (12.45)	862 (17.59)	
Other race	1,216 (7.17)	391 (6.23)	385 (8.29)	149 (6.06)	291 (8.01)	
Education (%)						< 0.001
Under high school	2,350 (15.10)	299 (6.47)	821 (16.64)	275 (14.41)	955 (26.64)	
High school or equivalent	2,194 (20.83)	401 (13.73)	622 (20.43)	350 (24.13)	821 (29.71)	
Above high school	5,590 (64.08)	2,110 (79.80)	1,610 (62.92)	806 (61.46)	1,064 (43.65)	
Family PIR (%)						< 0.001
<1	1,786 (11.78)	222 (4.49)	460 (10.37)	303 (14.87)	801 (22.30)	
≥1	7,529 (88.22)	2,395 (95.51)	2,303 (89.63)	1,032 (85.13)	1799 (77.70)	
BMI, n (%)						< 0.001
<25	2,715 (26.95)	847 (29.77)	743 (25.15)	360 (24.42)	765 (26.38)	
≥25	7,373 (73.05)	1,954 (70.23)	2,294 (74.85)	1,062 (75.58)	2063 (73.62)	
Drinking, n (%)						< 0.001
Never	1,452 (14.47)	442 (15.22)	601 (20.10)	128 (8.73)	281 (10.95)	
Mild	3,988 (54.04)	1,358 (61.98)	1,114 (52.54)	627 (53.55)	889 (43.17)	
Moderate	2,149 (28.79)	448 (21.45)	502 (25.14)	386 (34.62)	813 (40.74)	
Heavy	229 (2.70)	33 (1.35)	38 (2.22)	37 (3.10)	121 (5.14)	
Hypertension, n (%)	1,974 (17.70)	379 (12.67)	775 (24.91)	189 (13.18)	631 (20.07)	< 0.001
Dentition status, n (%)						< 0.001
Non-functional	1,896 (13.12)	168 (3.75)	657 (16.05)	174 (8.99)	897 (26.42)	
Functional	8,251 (86.88)	2,644 (96.25)	2,402 (83.95)	1,259 (91.01)	1,946 (73.58)	
Dental floss, n (%)						< 0.001
No	3,207 (27.72)	573 (19.70)	961 (27.59)	420 (26.85)	1,253 (40.36)	
Yes	6,861 (72.28)	2,224 (80.30)	2,074 (72.41)	1,004 (73.15)	1,559 (59.64)	
Glycemic status, n (%)						< 0.001
Normoglycemia	4532 (50.91)	1,568 (59.93)	1,092 (41.80)	787 (60.02)	1,085 (41.81)	
Prediabetes	3,956 (36.12)	948 (31.92)	1,305 (39.76)	482 (30.33)	1,221 (42.00)	
Diabetes mellitus	1,659 (12.97)	296 (8.15)	662 (18.44)	164 (9.65)	537 (16.19)	
Antihypertensive agents, n (%)	404 (12.34)	78 (8.57)	107 (8.50)	70 (20.25)	149 (17.95)	< 0.001
Antidiabetic agents, n (%)	1,026 (56.20)	265 (61.59)	356 (52.24)	124 (68.57)	281 (50.43)	< 0.001

Data are presented as the mean (SD) or number (%), as appropriate. Number of participants with missing data: BMI (n=59); smoking (n=5); drinking (n=2329); PIR (n=832); education (n=13), hypertension (n=714), and dental floss (n=79).

PIR, poverty income ratio; BMI, body mass index; TSE, tobacco smoke exposure.

### 3.2 Associations between periodontitis and TSE and glycemic status

**Table 2** shows the associations between periodontitis and TSE and the risk of prediabetes or DM, adjusted for potential confounders (in Model 3). When the participants with no TSE and no periodontitis were used as a reference, a significantly higher risk of prediabetes was found in participants with both TSE and periodontitis (OR=1.35; 95%CI: 1.14, 1.59, *P* =0.001). The results showed that participants with periodontitis solely and those with both TSE and periodontitis were independently associated with a 63% (OR=1.63; 95%CI: 1.28, 2.08, *P <*0.001) and 61% (OR=1.61; 95%CI: 1.23, 2.10, *P <*0.001) increased risk of DM, respectively. **Supplementary Table S1** shows the joint effect of TSE and periodontitis on glycemic status when participants were separated into four groups based on the status of periodontitis. Among the participants with TSE, a significantly higher risk of prediabetes or DM was found among those with moderate or severe periodontitis.

**Table 2:** Odds ratios with 95% confidence intervals for glycemic status upon co-exposure to TSE and periodontitis.

Glycemic status	Model 1		Model 2		Model 3	
	OR (95% CI)	*P*-value	OR (95% CI)	*P*-value	OR (95% CI)	*P*-value
Prediabetes vs normoglycemia						
No TSE and no periodontitis	Ref		Ref		Ref	
Periodontitis and no TSE	1.98 (1.76-2.22)	<0.001	1.39 (1.23-1.57)	<0.001	1.14 (0.98-1.34)	0.095
TSE and no periodontitis	1.01 (0.88-1.16)	0.856	1.22 (1.06-1.41)	0.006	1.15 (0.96-1.38)	0.124
TSE and periodontitis	1.86 (1.66-2.09)	<0.001	1.66 (1.47-1.88)	<0.001	1.35 (1.14-1.59)	0.001
Diabetes mellitus vs normoglycemia						
No TSE and no periodontitis	Ref		Ref		Ref	
Periodontitis and no TSE	3.21 (2.74-3.76)	<0.001	2.01 (1.69-2.38)	<0.001	1.63 (1.28-2.08)	<0.001
TSE and no periodontitis	1.10 (0.90-1.36)	0.354	1.48 (1.18-1.84)	0.001	1.24 (0.91-1.69)	0.177
TSE and periodontitis	2.62 (2.23-3.08)	<0.001	2.27 (1.91-2.70)	<0.001	1.61 (1.23-2.10)	<0.001

OR, odds ratio; CI, confidence interval; TSE, tobacco smoke exposure.

Model 1: crude ORs, no adjustment.

Model 2: adjusted for age and sex.

Model 3: adjusted for age, sex, race, education, body mass index, alcohol intake, family poverty income ratio, total cholesterol, triglyceride, hypertension, dentition status, and dental floss.

We also evaluated the exclusive effects of TSE and periodontitis on glycemic status without considering their respective mutual effects (**Supplementary Table S2**). Compared with participants with no TSE, those with TSE had an increased risk of prediabetes. The participants with periodontitis exhibited a positive association with the risk of prediabetes or DM compared to those without periodontitis. **Table 3** shows the reclassification of the effects of periodontitis and TSE on glycemic status in their respective various states. The results indicated that periodontitis was positively associated with the risk of prediabetes or DM in participants with TSE and DM in participants with no TSE.

**Table 3:** Risk reclassification of glycemic status based on TSE and periodontitis.

	Prediabetes		Diabetes mellitus	
	OR (95% CI)	P-value	OR (95% CI)	P-value
Scenario 1				
No TSE				
No periodontitis	Ref		Ref	
Periodontitis	1.17 (0.99-1.38)	0.065	1.80 (1.40-2.32)	<0.001
TSE				
No periodontitis	Ref		Ref	
Periodontitis	1.24 (1.03-1.50)	0.025	1.47 (1.09-1.98)	0.011
Scenario 2				
No periodontitis				
No TSE	Ref		Ref	
TSE	1.01 (0.82-1.24)	0.906	1.23 (0.88-1.72)	0.223
Periodontitis				
No TSE	Ref		Ref	
TSE	1.10 (0.92-1.30)	0.294	0.88 (0.69-1.11)	0.273

OR, odds ratio; CI, confidence interval; TSE, tobacco smoke exposure.

Model was adjusted for age, sex, race, education, body mass index, alcohol intake, family poverty income ratio, total cholesterol, triglyceride, hypertension, dentition status, and dental floss.

Scenario 1: effect of periodontitis on glycemic status between TSE groups; scenario 2: effect of TSE on glycemic status between periodontitis groups.

### 3.3 Subgroup analysis

**Figure 2:**
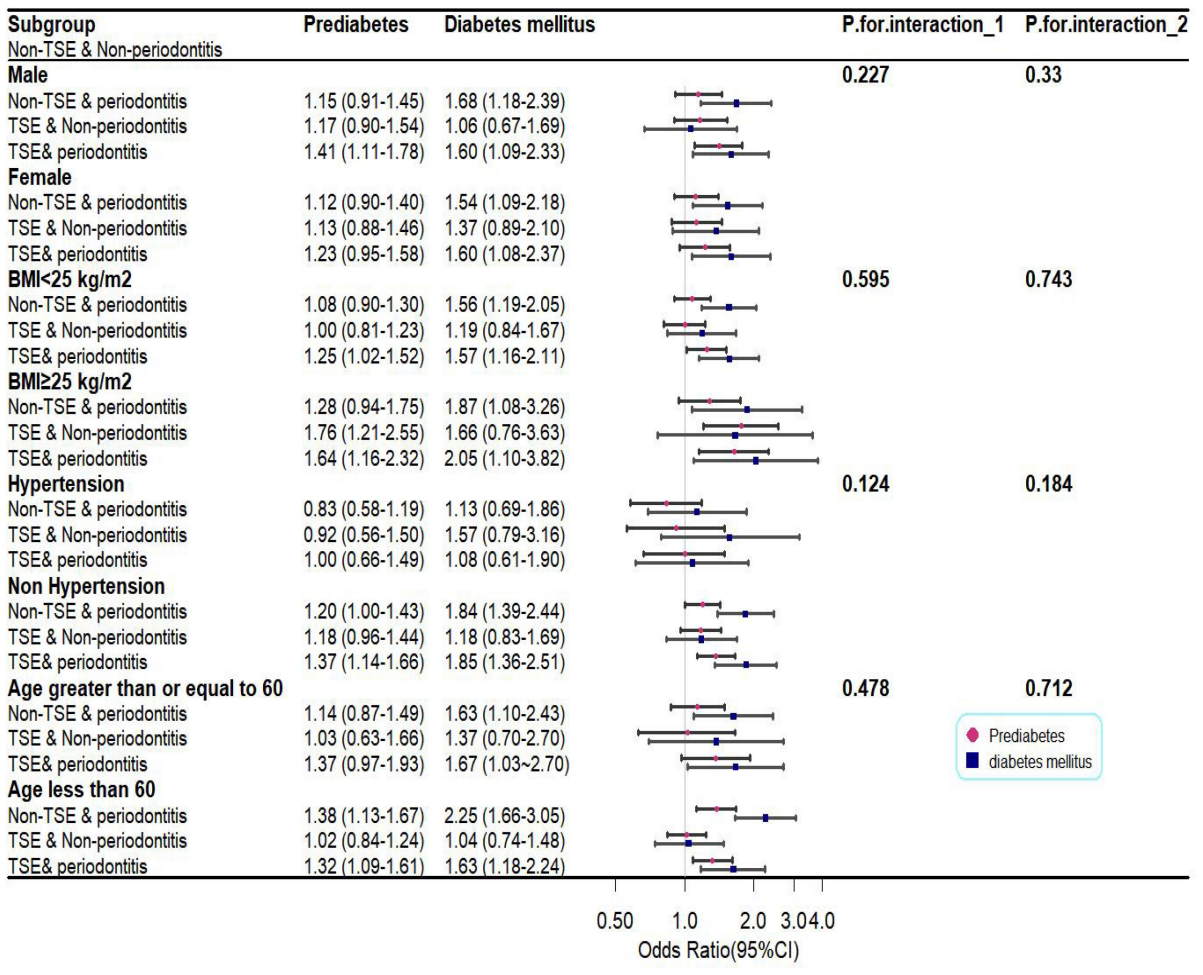
Plot of subgroup analysis for the combined effects of periodontitis and TSE on glycemic status. *P*_1_ and *P*_2_ represent, for prediabetes and diabetes mellitus, the interaction of the combined effects of periodontitis and TSE with confounders.

To discover whether the combined effects of periodontitis and TSE on glycemic status were potentially influenced by confounders, stratified analyses were conducted on age, sex, BMI, and hypertension. The results are displayed in **Figure 2**. Unlike in the whole population, the joint effect of periodontitis and TSE was not associated with an increased chance of prediabetes in the female, hypertension, and age ≥ 60 years groups. However, participants with BMI≥ 25 and TSE solely, and participants who were younger than 60 years old and only had periodontitis, were more likely to have prediabetes. Interestingly, no significant differences were found in the individual or joint effects of periodontitis and TSE in patients with hypertension on either DM or prediabetes.

### 3.4 Sensitivity analysis

A sensitivity analysis was conducted to evaluate the robustness of the primary results from the logistics regression model. **Supplementary Table S3** demonstrated that the joint effects of periodontitis and TSE on glycemic status remained consistent after using the complete dataset (8,512 participants) without multiple imputations. The difference was that participants with periodontitis solely had an increased risk of developing prediabetes when multiple imputed analyses were used. In addition, we repeated the analysis after TSE reclassification, and a positive association between periodontitis and the risk of prediabetes and DM was observed in both active and passive smokers (**Supplementary Table S4**). The results of analyses of the data after accounting for medication use as a covariate showed that the joint effects of periodontitis and TSE were no longer associated with an increased risk of prediabetes or DM (**Supplementary Table S5**).

## 4 Discussion

To the best of our knowledge, this is the first report to explore the joint effects of periodontitis and TSE on glycemic status among American adults. Conclusively, TSE and periodontitis were shown to be independently associated with prediabetes, and a positive association between periodontitis and DM was also observed. Moreover, a significant association between the joint effect of periodontitis and TSE and both DM and prediabetes was detected.

Exposure to tobacco smoke, including active smoking and passive smoking, has been implicated in the development of diabetes and prediabetes, with a growing body of research highlighting the association between these conditions and smoking. For example, one study (17) divided participants into two groups based on the passive smoking status. It was eventually observed that the prevalence of impaired glucose tolerance (IGT) was higher in passive smokers compared to non-smokers. In addition, results also indicated that passive smoking for >10 years increased the risk of impaired fasting glucose (IFG), IGT, and DM. A recent study found that non-smokers exposed to environmental tobacco smoke outside the home were positively associated with DM, particularly in those over 60 years of age and in women (18). Active smoking has also been linked to an increased risk of both prediabetes and DM (19, 20). A cross-sectional analysis (20) of healthy adults showed that current smokers (21) had a higher risk of prediabetes compared to never-smokers, with a dose-response relationship observed as the amount of smoking exposure increased. This suggests that smoking may accelerate the progression from normoglycemia to IGT, possibly by inducing insulin resistance and increasing the risk of DM. It is important to emphasize that quitting smoking does not immediately reduce the risk of DM. Studies have shown that the risk of DM increases in the first 2 years of smoking cessation, and decreases rapidly thereafter (22). The weight gain that frequently occurs after smoking abstinence could be a plausible hypothesis for the increased risk of developing DM (20).

The bidirectional prospective association between an individual’s periodontitis and the risk of prediabetes or DM was widely evaluated in a meta-analysis of cohort studies (23). The findings of this meta-analysis suggested the summary relative risk (SRR) for incident DM was higher in individuals with periodontitis than in individuals without periodontitis, and an increased SRR for incident periodontitis was also found in individuals with diabetes compared to individuals without diabetes. Consistent with our findings, evidence from a cross-sectional study suggested that periodontitis was associated with a prediabetic condition and it identified a statistically significant association between clinical attachment loss (CAL) and HbA1c (24). In individuals with uncontrolled diabetes, the most predominant alterations are a weakened immune response and increased vulnerability to infections, leading to the development of periodontitis. Hong et al. (25) found higher IFG in a prediabetic condition leads to a higher risk of developing periodontitis. The postulated mechanism linking periodontitis and glycemic status may be increased inflammation in the periodontal tissues. On the one hand, the serum levels of C-reactive protein (CRP) were higher in patients with prediabetes compared to individuals with normal glucose levels, and inflammation was not supplemented after developing diabetes (26). Periodontitis appears to occur in hyperglycemia as a result of low-grade systemic inflammation. It has also been shown that serum HbA1c levels were positively correlated with CRP levels and periodontal parameters in patients before diabetes onset (27). On the other hand, inflamed periodontal tissues induce local immune and inflammatory responses, leading to increased secretion of inflammatory cytokines and triggering upregulation of systemic inflammation (28), which in turn lead to impaired insulin signaling and insulin resistance that accelerate the deterioration of diabetes.

In this study, taking advantage of the objectivity of cotinine as the definition of TSE and a representative multi-stage design sample, we not only found a substantial joint effect of periodontitis and TSE on prediabetes or DM but also assessed the effect of both on glycemic status in their respective various states. The results showed that participants with periodontitis were positively associated with prediabetes or DM with TSE, and with DM with no TSE. Moreover, a positive association between periodontitis and the risk of prediabetes and DM was observed in both active and passive smokers, suggesting that smoking may contribute to exacerbating the deleterious effect of periodontitis on glycemic control. The results of the analysis from one study (29) that recruited 128 participants showed a significant difference in the severity of periodontitis between the smokers without DM and non-smokers without DM and between the non-smokers without DM and smokers with DM. Smoking seemed to cause the normally distributed severity spectrum of periodontitis to move toward the moderate stage, and a marked shift toward the severe stage was observed when smoking was combined with DM. In our study, regardless of stage, participants with periodontitis solely were not associated with prediabetes but were significantly associated with DM. Notably, an increased risk of prediabetes or DM was found when TSE was combined with moderate or severe periodontitis. Smoking repeatedly exposes periodontal tissue to toxic chemicals that sustain high levels of severe inflammation, and the effects are rapid and dramatic. In contrast, the relationship between periodontitis and DM develops gradually in a slow inflammatory process. This could explain the inherently positive association between periodontitis and diabetes regardless of TSE. Therefore, it was safe to assume that periodontitis was positively associated with a risk of DM rather than prediabetes and that smoking might alter or aggravate this harm.

The half-life of cotinine, derived from nicotine, can be up to 19–20 h, which reflects the degree of TSE over the previous 3–4 days (30). To distinguish smokers from non-smokers, Benowitz et al. (31) suggested a suitable cutoff of approximately 10 ng/mL. Unless there is prolonged exposure to high concentrations of tobacco smoke or active smoking, the value of serum cotinine concentration should be below the cut-off value. This could explain why the combined effect of periodontitis and TSE on prediabetes was not observed in women, those with hypertension, and those aged ≥60 years, due to the low frequency of smoking in these groups. We assume that the local inflammation caused by periodontitis was not sufficient to induce abnormal blood glucose or that the duration of it was not too long. In addition, if people with periodontitis smoke or are continuously exposed to high concentrations of tobacco smoke in a period of time, the risk of prediabetes will increase, and the harm of periodontitis to diabetes will be aggravated. Our findings thus underline the importance of avoiding exposure to tobacco smoke or quitting smoking for the management of glycemic status, especially for people with DM.

Our research had a number of strengths. In particular, this was the first study to comprehensively explore the joint association between periodontitis and TSE and glycemic status, which could potentially provide more precise estimates of their relationship. Additionally, participants were selected from the NHANES dataset that reflected the overall situation in the USA, thus, the results were more persuasive. Furthermore, in patients with periodontitis, our study demonstrated that TSE could result in blood glucose abnormalities and increase the risk of DM. These findings are worthy of reference for the management of the glycemic status of patients with periodontitis, highlighting the importance of avoiding constant exposure to tobacco smoke or quitting smoking. Several potential limitations warrant attention in this study. First, NHANES was a cross-sectional survey that temporally recorded the levels of cotinine and FBG, thus variation was not continuously observed. Therefore, it was not only impossible for us to determine causality but also difficult to exclude reverse causality. Second, because a definition of periodontitis has not been universally established, various diagnostic criteria for periodontitis may lead to different estimated effects. Finally, the participants of this study were almost all Caucasian, and studies in other populations are encouraged to further validate the associations.

## 5 Conclusion

Periodontitis and TSE synergistically increase the risk of incident DM or prediabetes, and the deleterious effect of periodontitis on glycemic control can be reduced by smoking abstinence. The findings highlight the importance of avoiding constant exposure to tobacco smoke or quitting smoking for the management of glycemic status of patients with moderate or severe periodontitis.

## Data Availability

The raw data supporting the conclusions of this article will be made available by the authors, without undue reservation.
