# Supplementary material for: Joint association between tobacco smoke exposure and periodontitis and glycemic status
Source: Front Endocrinol (Lausanne). 2025 Apr 7;16:1539955. doi: 10.3389/fendo.2025.1539955 (PMC12010096; doi:10.3389/fendo.2025.1539955)
Supplement: Supplementary file 1 [file Table1.docx]

| **Table S1** OR (95% CIs) of glycemic status upon co-exposure stratified by the TSE and periodontitis when the status of periodontitis are further separated into four groups. | | | | | |
| --- | --- | --- | --- | --- | --- |
| Subgroups | Prediabetes vs Normoglycaemia |  |  | Diabetes mellitus vs Normoglycaemia |  |
|  | OR (95% CI) | P value |  | OR (95% CI) | P value |
| Non-TSE & Non-periodontitis | Ref |  |  | Ref |  |
| Non-TSE & Mild periodontitis | 0.66 (0.42-1.04) | 0.073 |  | 1.81 (1.02-3.19) | 0.041 |
| Non-TSE & Moderate periodontitis | 1.17 (0.99-1.38) | 0.067 |  | 1.57 (1.21-2.02) | 0.001 |
| Non-TSE & Severe periodontitis | 1.21 (0.89-1.63) | 0.222 |  | 1.91 (1.28-2.85) | 0.002 |
| TSE & Non-periodontitis | 1.14 (0.95-1.37) | 0.170 |  | 1.25 (0.91-1.71) | 0.163 |
| TSE & Mild periodontitis | 1.23 (0.78-1.94) | 0.382 |  | 1.99 (0.97-4.06) | 0.060 |
| TSE & Moderate periodontitis | 1.33 (1.11-1.60) | 0.002 |  | 1.46 (1.09-1.95) | 0.011 |
| TSE & Severe periodontitis | 1.32 (1.01-1.71) | 0.039 |  | 2.01 (1.39-2.92) | <0.001 |
| OR: odds ratio; CI: Confidence interval; TSE, tobacco smoke exposure Model was adjusted for age, sex, race, education, body mass index, alcohol intake, family poverty income ratio, total cholesterol, triglyceride, hypertension, dentition.status, dental.floss. | | | | | |

| **Table S2** OR (95% CIs) for glycemic status upon individual exposure stratified by the TSE and periodontitis. | | | | | | | | |
| --- | --- | --- | --- | --- | --- | --- | --- | --- |
| Glycemic status | Model 1 |  |  | Model 2 |  |  | Model 3 |  |
|  | OR (95% CI) | *P* value |  | OR (95% CI) | *P* value |  | OR (95% CI) | *P* value |
| Non-TSE | Ref |  |  | Ref |  |  | Ref |  |
| TSE |  |  |  |  |  |  |  |  |
| Prediabetes vs Normoglycaemia | 1.07 (0.99-1.17) | 0.105 |  | 1.28 (1.17-1.41) | <0.001 |  | 1.17 (1.03-1.34) | 0.015 |
| Diabetes mellitus vs Normoglycaemia | 1.04 (0.93-1.17) | 0.503 |  | 1.36 (1.20-1.54) | <0.001 |  | 1.10 (0.91-1.34) | 0.324 |
| Non-periodontitis | Ref |  |  | Ref |  |  | Ref |  |
| Periodontitis |  |  |  |  |  |  |  |  |
| Prediabetes vs Normoglycaemia | 1.91 (1.75-2.08) | <0.001 |  | 1.43 (1.30-1.57) | <0.001 |  | 1.16 (1.02-1.31) | 0.022 |
| Diabetes mellitus vs Normoglycaemia | 2.82 (2.50-3.19) | <0.001 |  | 1.88 (1.65-2.15) | <0.001 |  | 1.50 (1.24-1.82) | <0.001 |
| OR: odds ratio; CI: Confidence interval; TSE, tobacco smoke exposure Model 1: crude ORs, no adjustment. Model 2: adjusted for age, sex. Model 3: adjusted for age, sex, race, education, body mass index, alcohol intake, family poverty income ratio, total cholesterol, triglyceride, hypertension, dentition.status, dental.floss. | | | | | | | | |

| **Table S3** Sensitivity analyses of co-exposure for TSE and periodontitis in terms of data missing. | | | | | |
| --- | --- | --- | --- | --- | --- |
| Glycemic status | Sensitivity analysis1 | |  | Sensitivity analysis2 | |
|  | OR (95% CI) | *P* value |  | OR (95% CI) | *P* value |
| Prediabetes vs Normoglycaemia |  |  |  |  |  |
| Non-TSE & Non-periodontitis | Ref |  |  | Ref |  |
| Non-TSE & periodontitis | 1.13 (0.96-1.32) | 0.140 |  | 1.16 (1.02-1.32) | 0.021 |
| TSE & Non-periodontitis | 1.14 (0.95-1.37) | 0.159 |  | 1.11 (0.95-1.29) | 0.203 |
| TSE& periodontitis | 1.32 (1.11-1.56) | 0.002 |  | 1.34 (1.16-1.54) | <0.001 |
| Diabetes mellitus vs Normoglycaemia |  |  |  |  |  |
| Non-TSE & Non-periodontitis | Ref |  |  | Ref |  |
| Non-TSE & periodontitis | 1.67 (1.32-2.11) | <0.001 |  | 1.50 (1.24-1.82) | <0.001 |
| TSE & Non-periodontitis | 1.27 (0.94-1.72) | 0.119 |  | 1.24 (0.97-1.60) | 0.088 |
| TSE& periodontitis | 1.76 (1.36-2.28) | <0.001 |  | 1.57 (1.27-1.94) | <0.001 |
|  |  |  |  |  |  |
| Sensitivity analysis1 was performed among 8512 participants without any missing data; sensitivity analysis 2 were performed using multiple imputed analyses (5iterations) by Markov chain Monte Carlo method.  Number of missing: education(n=13), family poverty income ratio (n=829); BMI (n=52); hypertension (n=617); triglyceride (n=57); dental floss (n=67) | | | | | |

| **Table S4** OR (95% CIs) of glycemic status upon co-exposure stratified by the TSE and periodontitis after TSE reclassification. | | | | | | | | |
| --- | --- | --- | --- | --- | --- | --- | --- | --- |
| Glycemic status | Model 1 |  |  | Model 2 |  |  | Model 3 |  |
|  | OR (95% CI) | *P* value |  | OR (95% CI) | *P* value |  | OR (95% CI) | *P* value |
| Prediabetes vs Normoglycaemia |  |  |  |  |  |  |  |  |
| Non-TSE & Non-periodontitis | Ref |  |  | Ref |  |  | Ref |  |
| PS & Non-periodontitis | 1.16 (0.97-1.38) | 0.101 |  | 1.31 (1.10-1.58) | 0.003 |  | 1.17 (0.93-1.47) | 0.184 |
| AS & Non-periodontitis | 0.89 (0.74-1.07) | 0.204 |  | 1.15 (0.95-1.39) | 0.164 |  | 1.14 (0.89-1.45) | 0.296 |
| Non-TSE & periodontitis | 1.98 (1.77-2.22) | < 0.001 |  | 1.40 (1.24-1.58) | < 0.001 |  | 1.12 (0.96-1.32) | 0.146 |
| PS & periodontitis | 2.10 (1.81-2.45) | < 0.001 |  | 1.78 (1.52-2.09) | < 0.001 |  | 1.25 (1.01-1.55) | 0.042 |
| AS & periodontitis | 1.72 (1.50-1.96) | < 0.001 |  | 1.59 (1.38-1.83) | < 0.001 |  | 1.35 (1.11-1.64) | 0.003 |
| Diabetes mellitus vs Normoglycaemia |  |  |  |  |  |  |  |  |
| Non-TSE & Non-periodontitis | Ref |  |  | Ref |  |  | Ref |  |
| PS & Non-periodontitis | 1.25 (0.96-1.62) | 0.098 |  | 1.52 (1.15-2.00) | 0.003 |  | 1.08 (0.73-1.59) | 0.691 |
| AS & Non-periodontitis | 0.99 (0.75-1.30) | 0.391 |  | 1.44 (1.08-1.92) | 0.014 |  | 1.48 (0.98-2.22) | 0.061 |
| Non-TSE & periodontitis | 3.23 (2.76-3.78) | < 0.001 |  | 2.03 (1.71-2.40) | < 0.001 |  | 1.64 (1.28-2.08) | < 0.001 |
| PS & periodontitis | 3.58 (2.94-4.36) | < 0.001 |  | 2.88 (2.34-3.54) | < 0.001 |  | 1.76 (1.29-2.42) | < 0.001 |
| AS & periodontitis | 2.02 (1.67-2.44) | < 0.001 |  | 1.84  (1.50-2.25) | < 0.001 |  | 1.47 (1.08-2.02) | 0.016 |
| OR: odds ratio; CI: Confidence interval; TSE, tobacco smoke exposure; PS, passive smoking; AS, active smoking. Model 1: crude ORs, no adjustment. Model 2: adjusted for age, sex. Model 3: adjusted for age, sex, race, education, body mass index, alcohol intake, family poverty income ratio, total cholesterol, triglyceride, hypertension, dentition.status, dental.floss. | | | | | | | | |

| **Table S5** OR (95% CIs) of glycemic status upon co-exposure stratified by the TSE and periodontitis further adjusting for medication uses | | | | | |
| --- | --- | --- | --- | --- | --- |
| Glycemic status | Prediabetes vs Normoglycaemia | |  | Diabetes mellitus vs Normoglycaemia | |
|  | OR (95% CI) | *P* value |  | OR (95% CI) | *P* value |
| Non-TSE & Non-periodontitis | Ref |  |  | Ref |  |
| Non-TSE & periodontitis | 1.24 (0.71-2.16) | 0.449 |  | 2.04 (1.13-3.69) | 0.018 |
| TSE & Non-periodontitis | 0.91 (0.47-1.77) | 0.777 |  | 1.26 (0.62-2.54) | 0.526 |
| TSE& periodontitis | 1.09 (0.58-2.05) | 0.781 |  | 1.78 (0.93-3.41) | 0.080 |
| OR: odds ratio; CI: Confidence interval; TSE, tobacco smoke exposure Model was adjusted for age, sex, race, education, body mass index, alcohol intake, family poverty income ratio, total cholesterol, triglyceride, hypertension, dentition.status, dental.floss, anti-hypertensive agents, anti-diabetic agents . | | | | | |
